# Supplementary material for: The Genetic and Molecular Analyses of RAD51C and RAD51D Identifies Rare Variants Implicated in Hereditary Ovarian Cancer from a Genetically Unique Population
Source: Cancers (Basel). 2022 Apr 30;14(9):2251. doi: 10.3390/cancers14092251 (PMC9104874; doi:10.3390/cancers14092251)
Supplement: Supplementary file 1 [file cancers-14-02251-s001.zip › Suppl Figures.pdf]

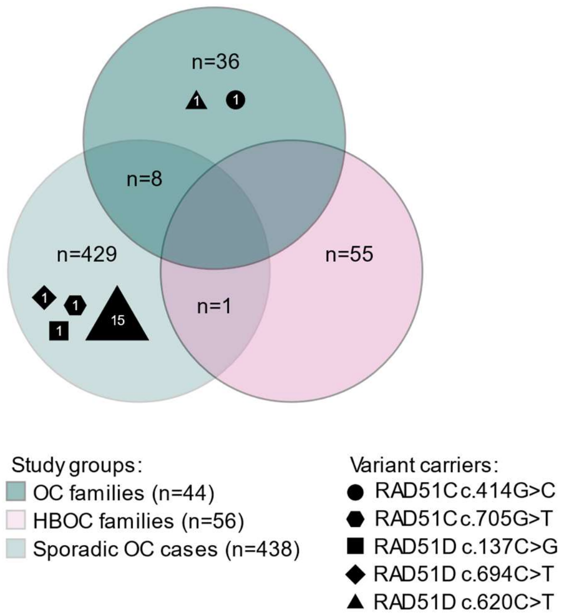

Figure S1. Venn diagram depicting relationship between carriers of candidate *RAD51C* or *RAD51D* variants in study groups investigated in phase II.
Each circle contains the total number (n) of cases investigated in ovarian cancer (OC) families, Hereditary Breast and Ovarian Cancer (HBOC) syndrome or sporadic disease study groups (see **Table S1**). The number of cases appearing in overlapping circles denote identical cases known to have been recruited to these study groups. The number inside each black symbol contains the carriers of each specific variant identified.

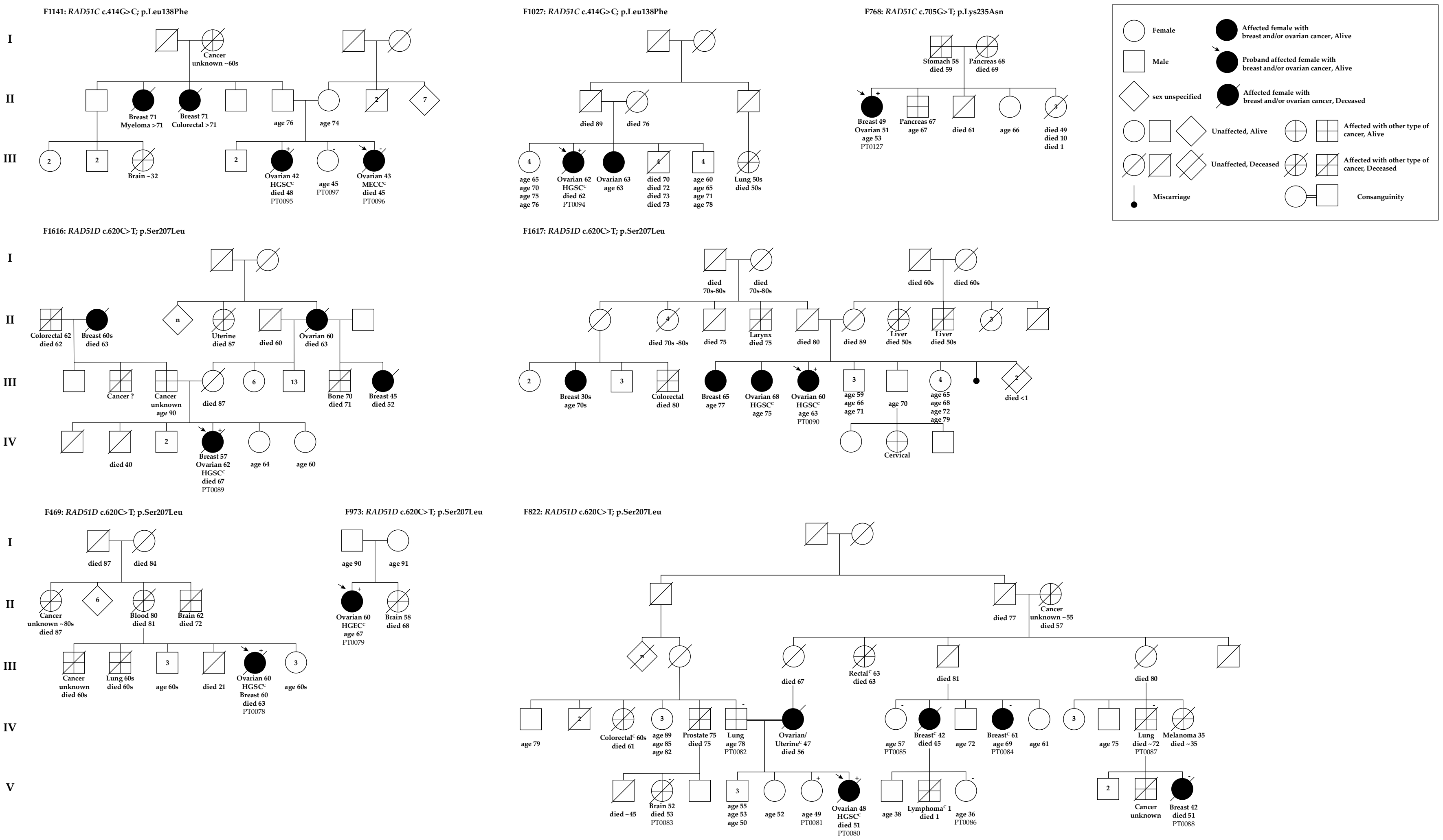

Figure S2. Pedigrees of selected index ovarian cancer cases carrying a candidate *RAD51C* or *RAD51D* variant.

Cases from family number F1616, F1617 and F1141 were part of phase I of the study, those from F822, and F1027 were part of phase II, and F469, F768 and F973 were part of phase III. Carrier status of index tested (arrow) and additional available family members are denoted by plus (carrier) or minus sign. All carriers were found in a heterozygous state. Age in years is shown with cancer diagnosis (HGSC: High-grade serous carcinoma of the ovary; MECC: Mixed high-grade endometrioid carcinoma of the ovary with clear cell) or death. Superscript C denotes histological subtypes that were confirmed by pathology reports or death certificates. Both pedigrees and case number tested were anonymized to protect the identities of the participants.

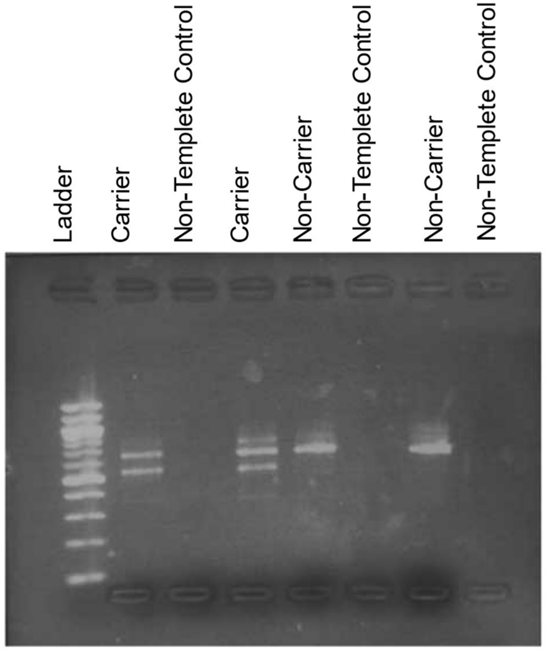

Figure S3. Uncropped gel electrophoresis of carrier- and non-carrier-derived lymphoblastoid cell lines (LCLs) of *RAD51C* c.705G>T.

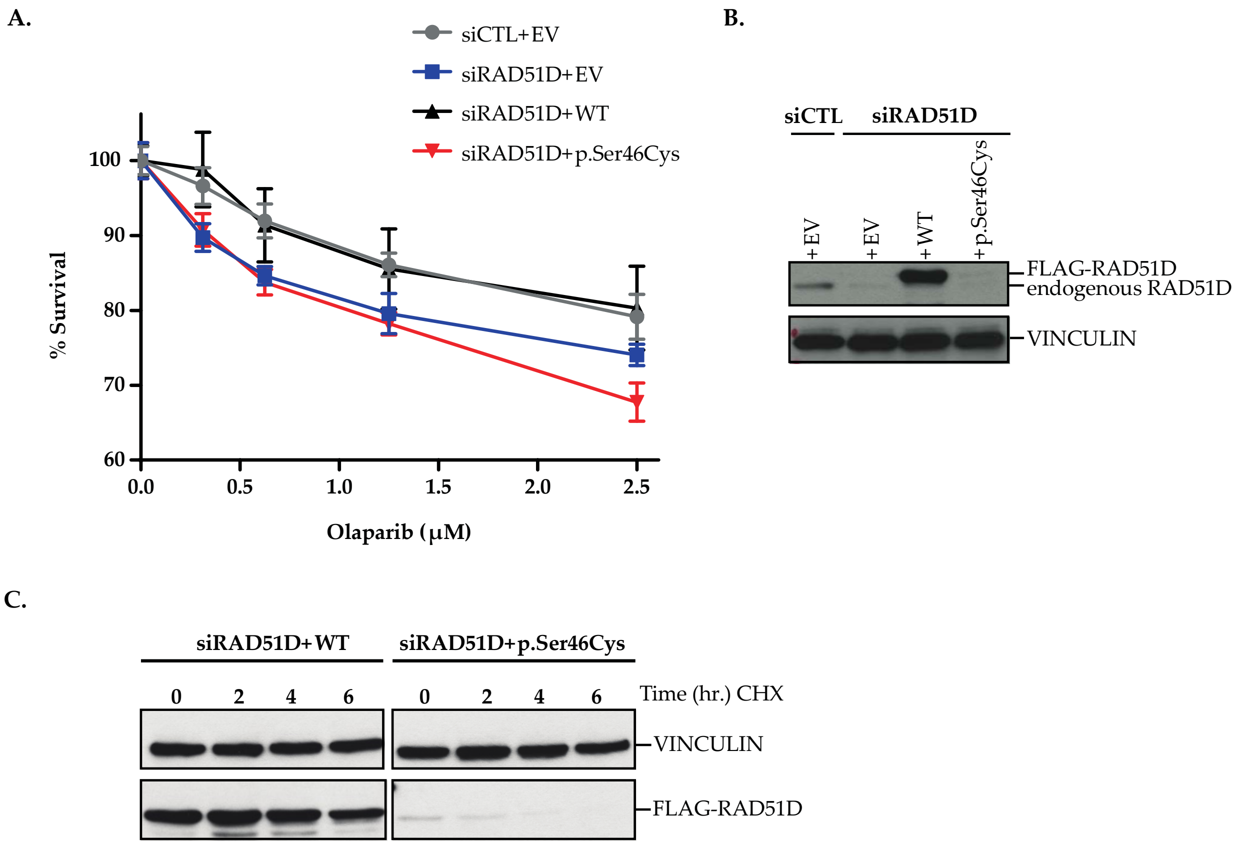

Figure S4. *In cellulo* functional characterization of the RAD51D p.Ser46Cys variant in the Hela cells
RAD51D-knockdown Hela cells, previously transfected with the empty vector (EV) or the indicated siRNA-resistant RAD51D constructs, including siRNA non-targeting controls (siCTL), were seeded in 96-well plates and exposed to increasing concentrations of olaparib. Cell viabilities were obtained from the 96-well plates post-treatment by quantification of surviving Hoechst-stained nuclei and represented in percentage of survival relative to the control (DMSO-treated) condition; (A) Viability curves contrasting the abilities of RAD51D wild-type and the p.Ser46Cys variant to rescue olaparib resistance to RAD51D-knockdown cells. Data is presented as the mean (± standard error mean [SEM]) from at least three independent experiments, each performed in triplicate; (B) RAD51D levels of after knockdown and re-expression in HeLa cells, with vinculin as loading control; (C) RAD51D-knockdown cells were transfected with FLAG-RAD51D wild-type or FLAG-RAD51D p.Ser46Cys. RAD51D protein levels were assessed at 2, 4, and 6 hours following addition of Cycloheximide (CHX) block. Vinculin was used as loading control.

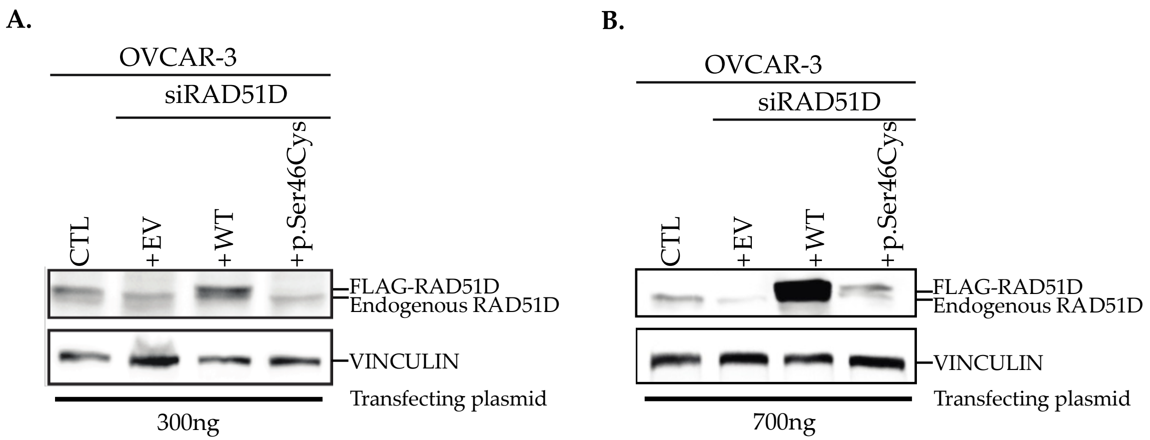

Figure S5. The RAD51D p.Ser46Cys variant impairs protein stability in the ovarian cancer cell line OVCAR-3.
Western blots of OVCAR-3 cells transfected with siRNA non-targeting control (siCTL) or targeting RAD51D (siRAD51D) and then complemented with FLAG-RAD51D constructs or empty vector (EV). (A) Cells transfected with 300ng of indicated plasmids; and (B) Cells transfected with 700ng of indicated plasmids. Vinculin was used as loading control.

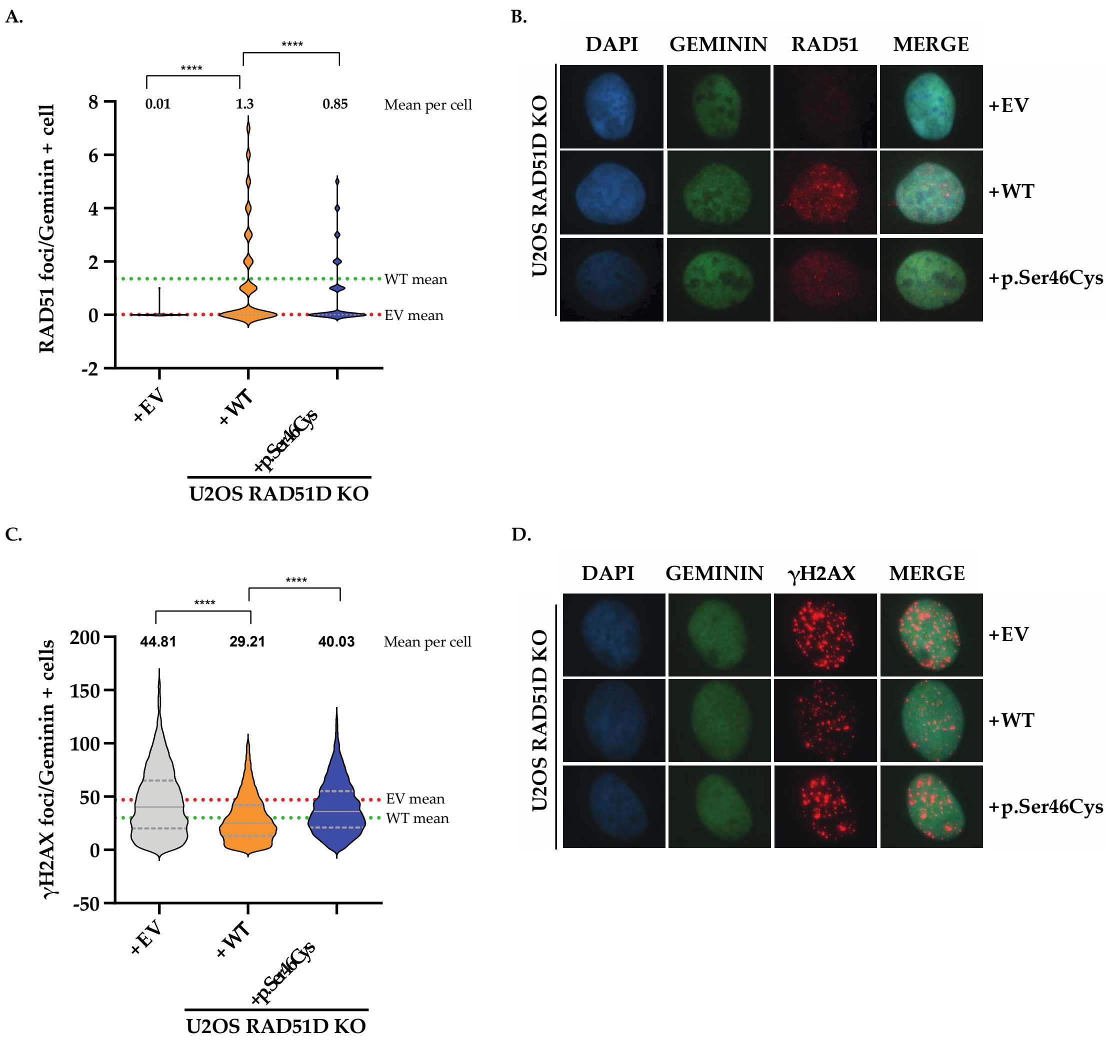

Figure S6. The RAD51D p.Ser46Cys variant impacts RAD51 and γH2AX foci formation in U2OS RAD51D knock-out (KO) cells.
(A-D) Immunofluorescence of U2OS RAD51D knock-out (KO) cells complemented with the indicated RAD51D constructs of RAD51D. Experiments have been performed in triplicate. Statistical significance was determined by Kruskal-Wallis test with Dunn's multiple comparison post-test (****$P<0.0001$) (A) Violin plots shows the number of RAD51D foci in Geminin-positive cells 4h after 5 Gray irradiation. (B) RAD51D immunofluorescence representative images. (C) Violin plot shows the number of γH2AX foci in Geminin-positive cells 4h after irradiation with 5Gray. (D) γH2AX immunofluorescence representative images. Dashed grey lines represent quartiles and the median is depicted with gray lines.

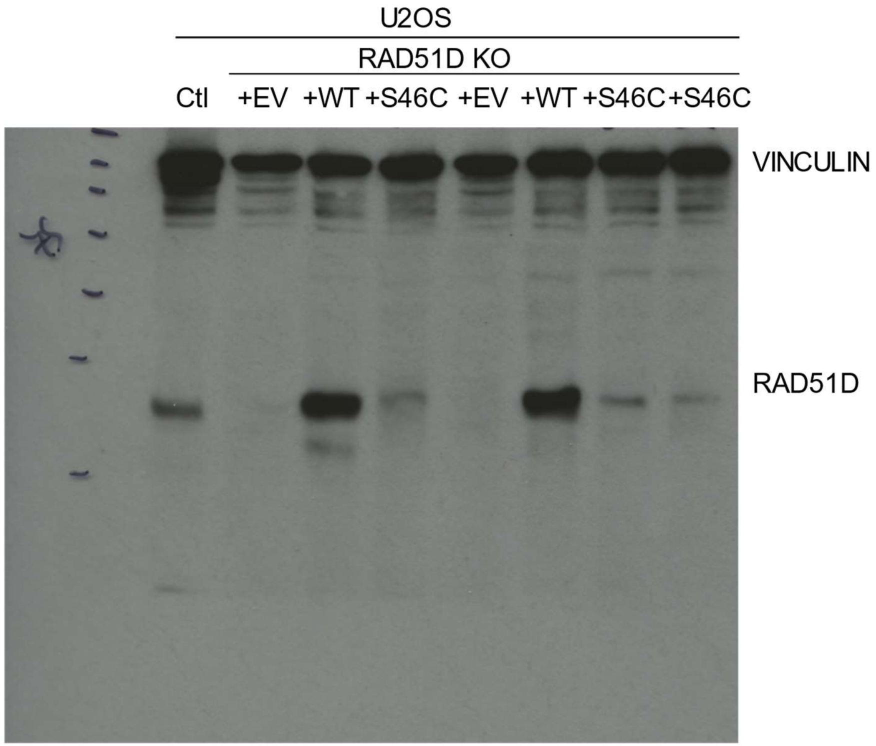

Figure S7. Uncropped western blot of U2OS RAD51D knock-out (KO) cells stably and complemented with wild-type (WT) or RAD51D p.Ser46Cys.

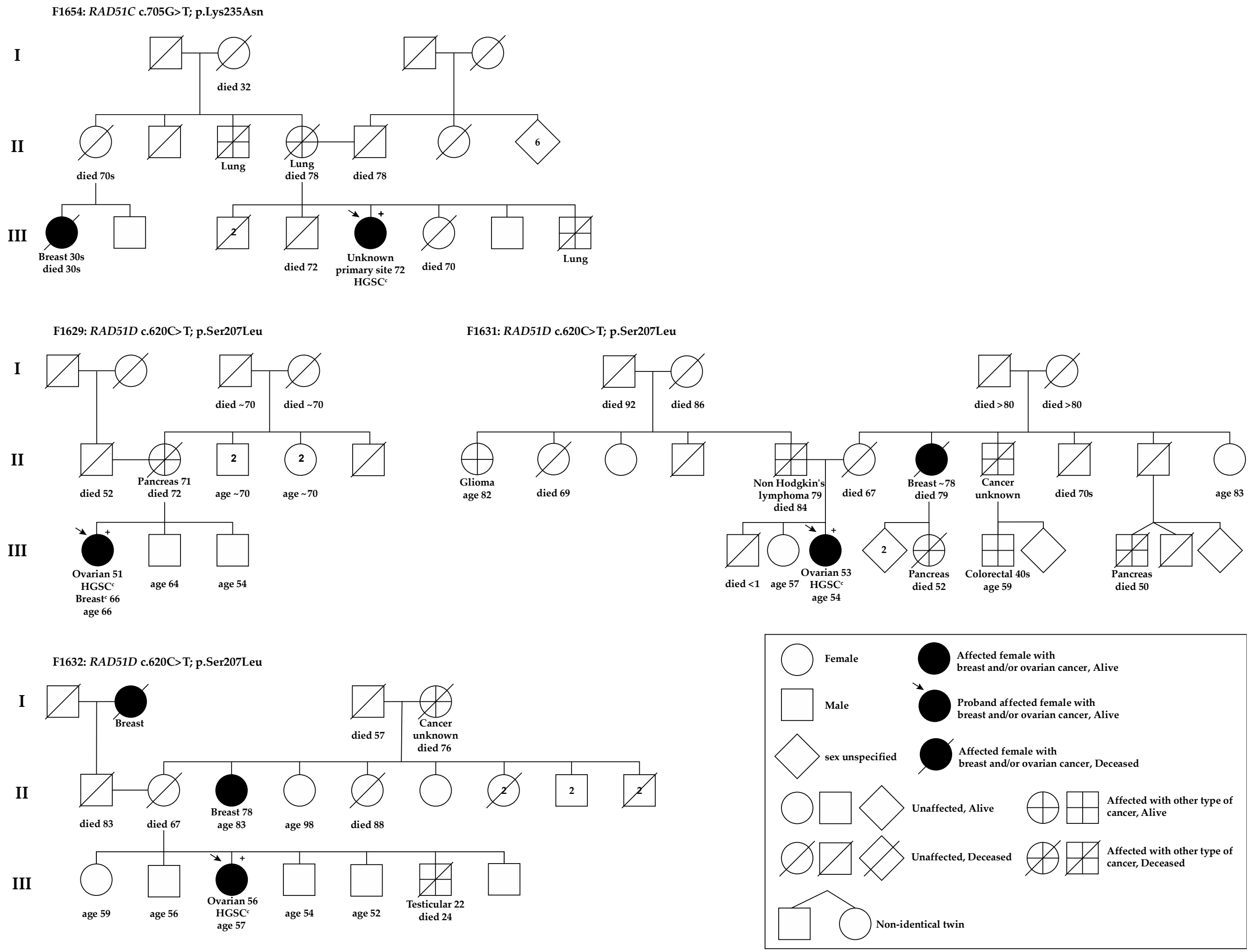

Figure S8. Additional pedigrees of ovarian cancer cases carrying a candidate *RAD51C* or *RAD51D* variant.
Carrier status of index case tested (arrow) denoted by a plus (carrier) or minus sign. All carriers were reported to be in heterozygous state. Age in years is shown with cancer diagnosis (HGSC: High-grade serous carcinoma of the ovary) or death. A review of pathology report of the index tested case in pedigree number F1654 confirmed a diagnosis of high-grade serous carcinoma of unknown origin (likely upper genital tract). Superscript C denotes histopathology was confirmed by pathology reports or death certificates. Pedigrees were anonymized to protect the identities of the participants.

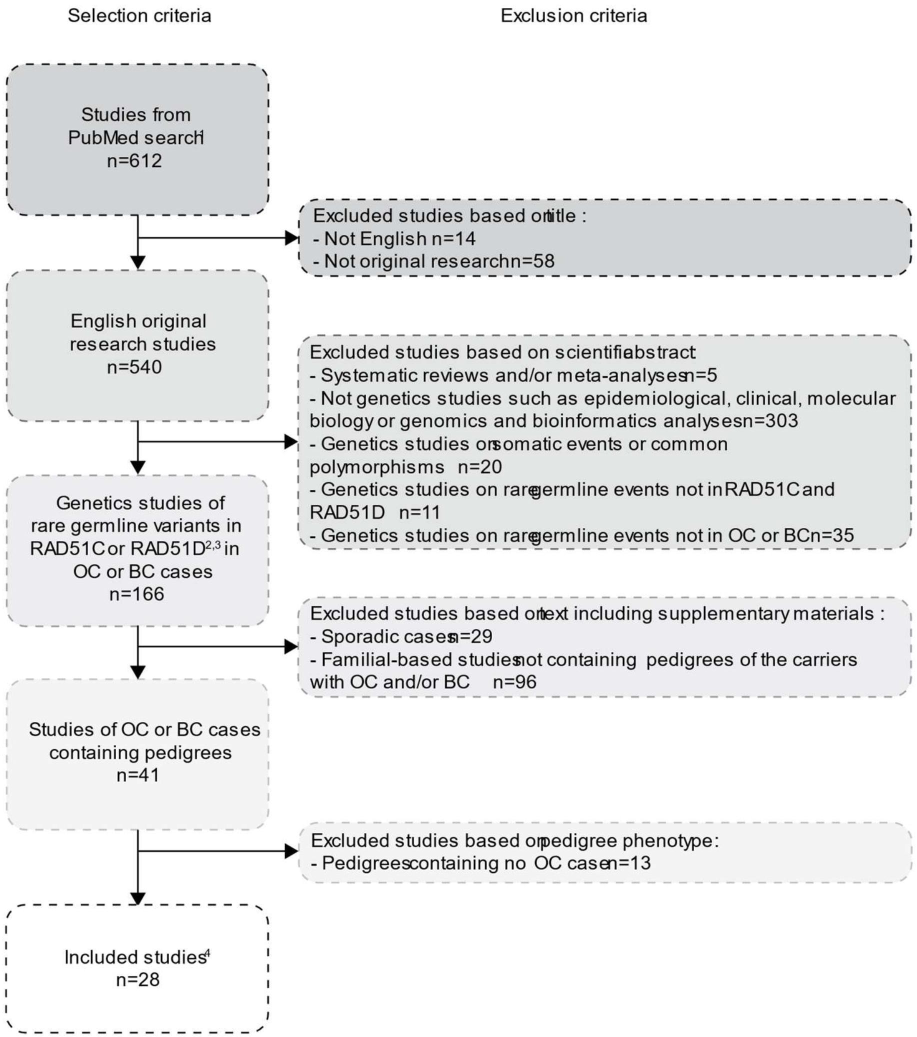

Figure S9. Schematic diagram of the criteria used for selecting rare *RAD51C* or *RAD51D* variants implicated in familial ovarian cancer from the published literature.

[1] PubMed database (pubmed.ncbi.nlm.nih.gov) was searched for articles up to October 2021 using the following terms: "RAD51C"[All Fields]; "FANCO"[All Fields]; "RAD51L2"[All Fields]; "RAD51D"[All Fields]; or "RAD51L3"[All Fields].

[2] Germline substitutions and small or large deletions or insertions variants were included.

[3] Germline variants classified as uncertain significance as reported by ClinVar and The American College of Medical Genetics and Genomics (ACMG) were excluded.

[4] Data from these studies appear in **Figure S5**.

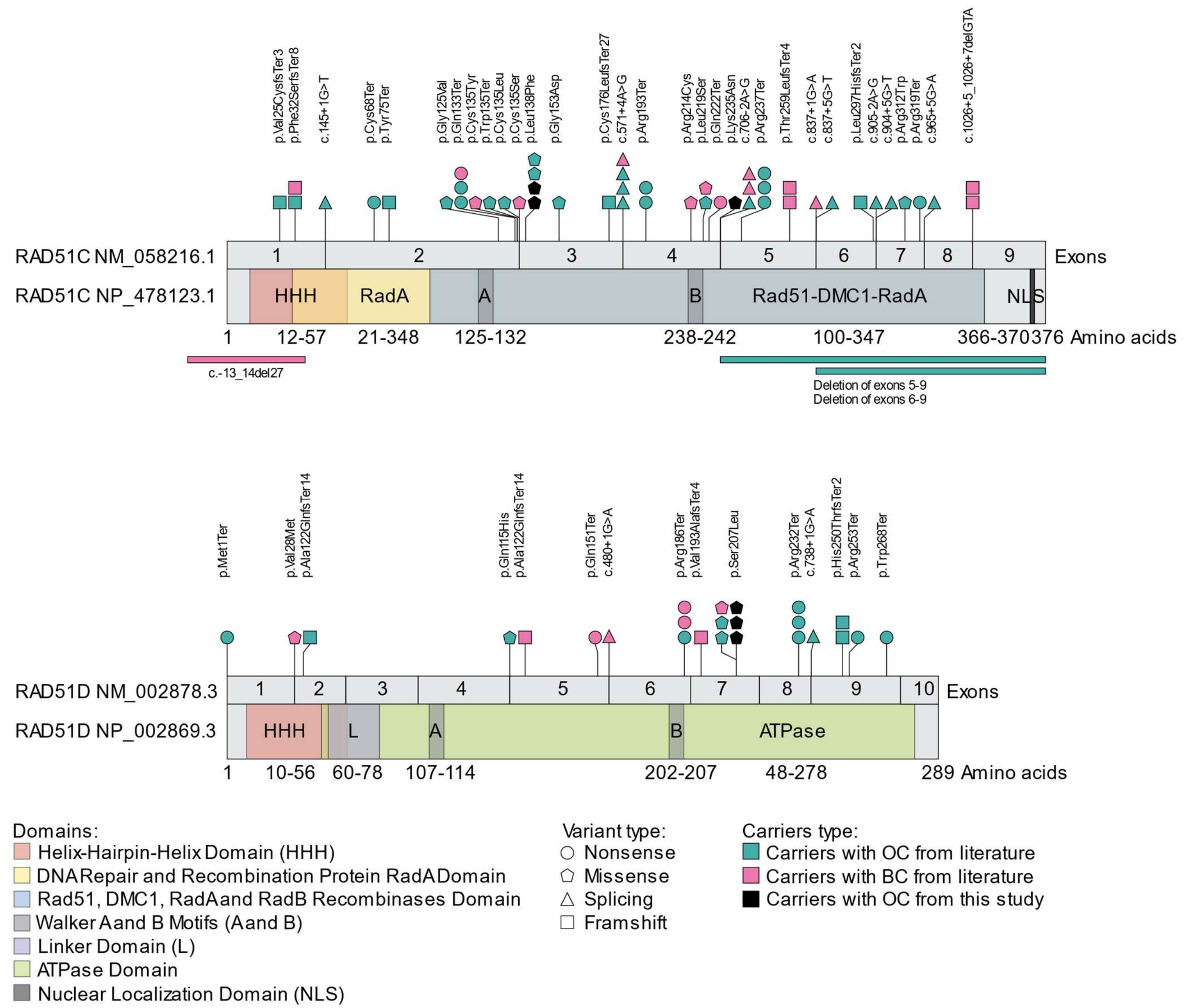

Figure S10. Lollipop figure of RAD51C and RAD51D functional protein domains with reported germline variants**.**
Lollipop depicting the location of all reported germline variants in published pedigrees of carriers with ovarian (OC) or breast cancer (BC) cases with a family history of OC in known protein domains of *RAD51C* at the mRNA (NM_058216.1) and protein (NP_478123.1) level (upper panel) and *RAD51D* at mRNA (NM_002878.3) and protein (NP_002869.3) level (lower panel) based on the NCBI Reference Sequence (RefSeq) database (ncbi.nlm.nih.gov/refseq/).
