# Supplementary material for: The Genetic and Molecular Analyses of RAD51C and RAD51D Identifies Rare Variants Implicated in Hereditary Ovarian Cancer from a Genetically Unique Population
Source: Cancers (Basel). 2022 Apr 30;14(9):2251. doi: 10.3390/cancers14092251 (PMC9104874; doi:10.3390/cancers14092251)
Supplement: Supplementary file 1 [file cancers-14-02251-s001.zip › Suppl Table S1.pdf]

Table S1. Description of French Canadian study groups investigated in this study.

Study group[1]	Participant investigated	Number of participants (or families) investigated	Age range in years (average ± standard deviation)	Characteristics of study group and pathogenic *BRCA1* or *BRCA2* variant carrier status at selection for this study (FC ancestry[1])	Purpose of the study group (study phase)	Source of genetic information (number of participants subjected to genotyping or sequencing method) in this study[3]	Source of participants[1]
Familial OC cases	OC	20 (17)	24-74 (52.6±11.3)	Families with at least two epithelial OC, fallopian tube or primary peritoneal carcinomas within first-, second- or third-degree relatives; at least one OC case tested negative for pathogenic *BRCA1* and *BRCA2* variants by FC mutation-panel or clinical multi-gene panel testing.	Identify candidate variants in familial OC cases (phase I)	WES (20)	RRCancer biobank or adult hereditary cancer clinics [1–11]
Sporadic early-onset OC cases	OC	53	24-49 (44.1±5.5)	High-grade serous ovarian carcinoma cases diagnosed <50 years, not selected for family history of OC cancer; tested negative for pathogenic *BRCA1* and *BRCA2* variants by FC mutation-panel or clinical multi-gene panel testing.	Identify candidate variants in sporadic OC cases (phase I)	WES (53)	RRCancer biobank [1,2,4–8,10–16]
OC families	OC	49 (44)	31-77 (52.2±9.4)	Additional families with at least two OC cases diagnosed with epithelial OC, fallopian tube or primary peritoneal carcinomas within first-, second- or third-degree relatives within the same family branch; 71% of index OC cases tested positive for pathogenic *BRCA1* and *BRCA2* variants by FC mutation-panel or clinical multi-gene panel testing [5,7].	Determine carrier frequency of candidate variants in two OC case families (phase II)	WES (25) and TaqMan® genotyping (24)	RRCancer biobank [1–10,13,14]
HBOC families	OC or BC[2]	14 OC or<br><br>42 BC (56)	OC: 24-73 (49.6±13.7)<br>BC: 30-61 (45.7±7.2)	Families with only one epithelial OC, fallopian tube or primary peritoneal carcinomas case and at least two invasive breast cancer cases (diagnosed <65 years) within first-, second- or third-degree relatives within the same family branch; 54% of index OC/BC cases tested positive for pathogenic *BRCA1* and *BRCA2* variants by FC mutation-panel or clinical multi-gene panel testing [5,7].	Determine carrier frequency of candidate variants in HBOC families (phase II)	WES (6) and TaqMan® genotyping (50)	RRCancer biobank [1,2,11,3–10]
Sporadic OC cases	OC	438	24-91 (60.8±10.5)	Epithelial OC, fallopian tube or primary peritoneal carcinomas cases (includes high-grade serous, high grade endometrioid, low-grade, ungraded serous or mixed subtypes tumours containing serous component were included) not selected for age at diagnosis or family history of OC cancer; 12% tested positive for pathogenic *BRCA1* and *BRCA2* variants by FC mutation-panel testing [14].	Determine carrier frequency of candidate variants in sporadic OC cases (phase II)	WES (52) and TaqMan® genotyping (386)	RRCancer biobank [3,14,17]

Sequencing-based controls[3]	Control	1025	Gen3G - newborn; MNI (not available); CARTaGENE - 41-70 (55.2±8.6)	Gen3G controls: 433 newborns (sex not divulged) from mothers were recruited between 2010-2013 to study genetics of glucose regulation in gestation and growth.<br><br>MNI controls: 422 adult participants (202 females and 210 males) were recruited during different periods of time to study genetics of neurodegenerative or neurological disorders (311 affected; 11 unaffected and remaining unknown).<br><br>CARTaGENE controls: 170 adult participants (52 females and 118 males) were recruited between 2009–2014 to study the causes of chronic diseases.<br><br>Personal and family history of cancer, and pathogenic *BRCA1* and *BRCA2* variant carrier status unknown for all controls.	Determine carrier frequency of candidate variants in population-matched controls from DNA sequencing data (phase II)	WGS - Gen3G (433); WES - MNI (422) and CARTaGENE (170)	Gen3G project; MNI project; and CARTaGENE [17–23]
Genotyping-based controls[3]	Control	8493	39-71 (54.4±7.6)	CARTaGENE controls comprised of 4625 females and 3868 males recruited as described in sequencing-based controls: personal and family history of cancer, and pathogenic *BRCA1* and *BRCA2* variant carrier status unknown.	Determine carrier frequency of candidate variants in population-matched controls from genotyping-based data (phase II)	SNP array genotyping (100)	CARTaGENE [19–21]
Additional OC cases	OC	538	69% known: 24-89 (59.2±11.9)	Epithelial ovarian, fallopian tube or primary peritoneal carcinomas not categorized based on any defined inclusion criteria; pathogenic *BRCA1* and *BRCA2* variant carrier status unknown.	Identify additional carriers of candidate variants (phase III)	WES (44) and TaqMan® genotyping (494)	RRCancer biobank [24]

[1] All study groups contain participants recruited from the province of Quebec: familial ovarian cancer (OC) or invasive breast (BC) cases self-reported French Canadian (FC) ancestry [5,7]; the majority of sporadic OC (at least 88%) self-reported FC ancestry [14]; and additional OC cases self-reported FC ancestry by the Banque de tissus et données of the Réseau de recherche sur le cancer of the Fond de recherche du Québec – Santé (RRCancer biobank) or adult hereditary cancer clinics; Sherbrook University - glucose regulation in gestation and growth (Gen3G) controls included newborn whose mothers self-reported FC ancestry [18]; McGill University - Montreal Neurological Institute (MNI) controls were self-reported as FC ancestry [17,23]; and CARTaGENE defined FC status of controls based on being born in Quebec, having parents and all four grandparents born in Canada and having French as first language learned [19].

[2] BC case in closest familial relationship to OC was investigated where OC case was not available in the investigation of Hereditary Breast and Ovarian Cancer (HBOC) syndrome families study group.

[3] Genetic data derived from whole exome sequencing (WES) analyses or targeted TaqMan® genotyping assay for familial, sporadic or additional cases as described in Methods of this study; whole genome sequencing (WGS) analyses for Gen3G [18]; MNI [17]; and CARTaGENE [20–22] controls (sequencing-based controls); and three different single nucleotide (SNP) genotyping arrays analyses for CARTaGENE (genotyping-based controls): Affymetrix UK biobank Axiom® 2.0 gene chip (n=772) [19], Infinium® Global Screening Array (GSA) conducted in three phases (n=3159, 628, 3427) [19] and Infinium Omni 2.5 (n=510) [20,21].

References

1. Osher, D.J.; De Leeneer, K.; Michils, G.; Hamel, N.; Tomiak, E.; Poppe, B.; Leunen, K.; Legius, E.; Shuen, A.; Smith, E.; et al. Mutation analysis of RAD51D in non-BRCA1/2 ovarian and breast cancer families. *Br. J. Cancer* **2012**, doi:10.1038/bjc.2012.87.
2. Arcand, S.L.; Akbari, M.R.; Mes-Masson, A.M.; Provencher, D.; Foulkes, W.D.; Narod, S.A.; Tonin, P.N. Germline TP53 mutational spectrum in French Canadians with breast cancer. *BMC Med. Genet.* **2015**, doi:10.1186/s12881-015-0169-y.
3. Fierheller, C.T.; Guitton-Sert, L.; Alenezi, W.M.; Revil, T.; Oros, K.K.; Gao, Y.; Bedard, K.; Arcand, S.L.; Serruya, C.; Behl, S.; et al. A functionally impaired missense variant identified in French Canadian families implicates FANCI as a candidate ovarian cancer-predisposing gene. *Genome Med.* **2021**, *13*, doi:10.1186/S13073-021-00998-5.
4. Arcand, S.L.; Maugard, C.M.; Ghadirian, P.; Robidoux, A.; Perret, C.; Zhang, P.; Fafard, E.; Mes-Masson, A.M.; Foulkes, W.D.; Provencher, D.; et al. Germline TP53 mutations in BRCA1 and BRCA2 mutation-negative French Canadian breast cancer families. *Breast Cancer Res. Treat.* **2008**, doi:10.1007/s10549-007-9608-6.
5. Tonin, P.N.; Mes-Masson, A.-M.; Futreal, P.A.; Morgan, K.; Mahon, M.; Foulkes, W.D.; Cole, D.E.C.; Provencher, D.; Ghadirian, P.; Narod, S.A. Founder BRCA1 and BRCA2 Mutations in French Canadian Breast and Ovarian Cancer Families. *Am. J. Hum. Genet.* **1998**, doi:10.1086/302099.
6. Tonin, P.N.; Maugard, C.M.; Perret, C.; Mes-Masson, A.M.; Provencher, D.M. A review of histopathological subtypes of ovarian cancer in BRCA-related French Canadian cancer families. *Fam. Cancer* **2007**, doi:10.1007/s10689-007-9152-x.
7. Oros, K.K.; Ghadirian, P.; Greenwood, C.M.T.; Perret, C.; Shen, Z.; Paredes, Y.; Arcand, S.L.; Mes-Masson, A.M.; Narod, S.A.; Foulkes, W.D.; et al. Significant proportion of breast and/or ovarian cancer families of French Canadian descent harbor 1 of 5 BRCA1 and BRCA2 mutations. *Int. J. Cancer* **2004**, doi:10.1002/ijc.20406.
8. Oros, K.K.; Leblanc, G.; Arcand, S.L.; Shen, Z.; Perret, C.; Mes-Masson, A.M.; Foulkes, W.D.; Ghadirian, P.; Provencher, D.; Tonin, P.N. Haplotype analysis suggest common founders in carriers of the recurrent BRCA2 mutation, 3398delAAAAG, in French Canadian hereditary breast and/ovarian cancer families. *BMC Med. Genet.* **2006**, doi:10.1186/1471-2350-7-23.
9. Ancot, F.; Arcand, S.L.; Mes-Masson, A.M.; Provencher, D.M.; Tonin, P.N. Double PALB2 and BRCA1/BRCA2 mutation carriers are rare in breast cancer and breast-ovarian cancer syndrome families from the French Canadian founder population. *Oncol. Lett.* **2015**, doi:10.3892/ol.2015.3123.
10. Oros, K.; Ghadirian, P.; Maugard, C.; Perret, C.; Paredes, Y.; Mes-Masson, A.-M.; Foulkes, W.; Provencher, D.; Tonin, P. Application of BRCA1 and BRCA2 mutation carrier prediction models in breast and/or ovarian cancer families of French Canadian descent. *Clin. Genet.* **2006**, *70*, 320–329, doi:10.1111/J.1399-0004.2006.00673.X.
11. Alenezi, W.M.; Fierheller, C.T.; Revil, T.; Serruya, C.; Mes-Masson, A.-M.; Foulkes, W.D.; Provencher, D.; Haffaf, Z. El; Ragoussis, J.; Tonin, P.N. Case Review: Whole-Exome Sequencing Analyses Identify Carriers of a Known Likely Pathogenic Intronic BRCA1 Variant in Ovarian Cancer Cases Clinically Negative for Pathogenic BRCA1 and BRCA2 Variants. *Genes 2022, Vol. 13, Page 697* **2022**, *13*, 697, doi:10.3390/GENES13040697.

12. Tischkowitz, M.; Sabbaghian, N.; Hamel, N.; Pouchet, C.; Foulkes, W.D.; Mes-Masson, A.M.; Provencher, D.M.; Tonin, P.N. Contribution of the PALB2 c.2323C>T [p.Q775X] Founder mutation in well-defined breast and/or ovarian cancer families and unselected ovarian cancer cases of French Canadian descent. *BMC Med. Genet.* **2013**, doi:10.1186/1471-2350-14-5.
13. Tonin, P.N.; Mes-Masson, A.M.; Narod, S.A.; Ghadirian, P.; Provencher, D. Founder BRCA 1 and BRCA 2 mutations in French Canadian ovarian cancer cases unselected for family history. *Clin. Genet.* **1999**.
14. Belanger, M.H.; Dolman, L.; Arcand, S.L.; Shen, Z.; Chong, G.; Mes-Masson, A.M.; Provencher, D.; Tonin, P.N. A targeted analysis identifies a high frequency of BRCA1 and BRCA2 mutation carriers in women with ovarian cancer from a founder population. *J. Ovarian Res.* **2015**, doi:10.1186/s13048-015-0124-8.
15. Communal, L.; Medrano, M.; Sircoulomb, F.; Paterson, J.; Köbel, M.; Rahimi, K.; Hoskins, P.; Tu, D.; Lheureux, S.; Oza, A.; et al. Low junctional adhesion molecule-A expression is associated with an epithelial to mesenchymal transition and poorer outcomes in high-grade serous carcinoma of uterine adnexa. *Mod. Pathol.* **2020**, *33*, 2361–2377, doi:10.1038/s41379-020-0586-0.
16. Tischkowitz, M.; Xia, B.; Sabbaghian, N.; Reis-Filho, J.S.; Hamel, N.; Li, G.; Van Beers, E.H.; Li, L.; Khalil, T.; Quenneville, L.A.; et al. Analysis of PALB2/FANCN-associated breast cancer families. *Proc. Natl. Acad. Sci. U. S. A.* **2007**, doi:10.1073/pnas.0701724104.
17. Rivera, B.; Iorio, M. Di; Frankum, J.; Nadaf, J.; Fahiminiya, S.; Arcand, S.L.; Burk, D.L.; Grapton, D.; Tomiak, E.; Hastings, V.; et al. Functionally Null RAD51D Missense Mutation Associates Strongly with Ovarian Carcinoma. *Cancer Res.* **2017**, *77*, 4517–4529, doi:10.1158/0008-5472.CAN-17-0190.
18. Guillemette, L.; Allard, C.; Lacroix, M.; Patenaude, J.; Battista, M.C.; Doyon, M.; Moreau, J.; Ménard, J.; Bouchard, L.; Ardilouze, J.L.; et al. Genetics of Glucose regulation in Gestation and Growth (Gen3G): A prospective prebirth cohort of mother-child pairs in Sherbrooke, Canada. *BMJ Open* **2016**, doi:10.1136/bmjopen-2015-010031.
19. Awadalla, P.; Boileau, C.; Payette, Y.; Idaghdour, Y.; Goulet, J.P.; Knoppers, B.; Hamet, P.; Laberge, C. Cohort profile of the CARTaGENE study: Quebec's population-based biobank for public health and personalized genomics. *Int. J. Epidemiol.* **2013**, doi:10.1093/ije/dys160.
20. Hodgkinson, A.; Idaghdour, Y.; Gbeha, E.; Grenier, J.C.; Hip-Ki, E.; Bruat, V.; Goulet, J.P.; De Malliard, T.; Awadalla, P. High-resolution genomic analysis of human mitochondrial RNA sequence variation. *Science (80-.).* **2014**, doi:10.1126/science.1251110.
21. Hussin, J.G.; Hodgkinson, A.; Idaghdour, Y.; Grenier, J.C.; Goulet, J.P.; Gbeha, E.; Hip-Ki, E.; Awadalla, P. Recombination affects accumulation of damaging and disease-associated mutations in human populations. *Nat. Genet.* **2015**, doi:10.1038/ng.3216.
22. Peischl, S.; Dupanloup, I.; Foucal, A.; Jomphe, M.; Bruat, V.; Grenier, J.C.; Gouy, A.; Gilbert, K.J.; Gbeha, E.; Bosshard, L.; et al. Relaxed selection during a recent human expansion. *Genetics* **2018**, doi:10.1534/genetics.117.300551.
23. Gan-Or, Z.; Zhou, S.; Ambalavanan, A.; Leblond, C.S.; Xie, P.; Johnson, A.; Spiegelman, D.; Allen, R.P.; Earley, C.J.; Desautels, A.; et al. Analysis of functional GLO1 variants in the BTBD9 locus and restless legs syndrome. *Sleep Med.* **2015**, *16*, 1151–1155, doi:10.1016/J.SLEEP.2015.06.002.
24. Godard, B.; Foulkes, W.D.; Provencher, D.; Brunet, J.S.; Tonin, P.N.; Mes-Masson, A.M.; Narod, S.A.; Ghadirian, P. Risk factors for familial and sporadic ovarian cancer among French Canadians: A case-control study. *Am. J. Obstet. Gynecol.* **1998**, *179*, 403–410,

doi:10.1016/S0002-9378(98)70372-2.
